# Excellently balanced water-intercalation-type heat-storage oxide

**DOI:** 10.1038/s41467-022-28988-0

**Published:** 2022-03-17

**Authors:** Takuya Hatakeyama, Norihiko L. Okamoto, Satoshi Otake, Hiroaki Sato, Hongyi Li, Tetsu Ichitsubo

**Affiliations:** 1 Institute for Materials Research, Tohoku University, 2-1-1 Katahira, Aoba-ku, Sendai, 980-8577 Japan; 2 Graduate School of Engineering, Tohoku University 6-6-01 Aoba, Aramaki, Aoba-ku, Sendai, 980-8579 Japan; 3 Rigaku Corporation, 3-9-12 Matsubara-cho, Akishima, Tokyo 196-8666 Japan

**Keywords:** Energy storage, Thermodynamics

## Abstract

Importance of heat storage materials has recently been increasing. Although various types of heat storage materials have been reported to date, there are few well-balanced energy storage materials in terms of long lifetime, reversibility, energy density, reasonably fast charge/discharge capability, and treatability. Here we report an interesting discovery that a commonly known substance, birnessite-type layered manganese dioxide with crystal water (δ-type K_0.33_MnO_2 _⋅ *n*H_2_O), exhibits a water-intercalation mechanism and can be an excellently balanced heat storage material, from the above views, that can be operated in a solid state with water as a working pair. The volumetric energy density exceeds 1000 MJ m^−3^ (at *n* ~ 0.5), which is close to the ideally maximum value and the best among phase-change materials. The driving force for the water intercalation is also validated by the ab initio calculations. The proposed mechanism would provide an optimal solution for a heat-storage strategy towards low-grade waste-heat applications.

## Abstract (web summary)

There are few well-balanced heat storage materials up to date. Here, the authors report that δ-type K0.33MnO_2_ ∙ nH_2_O can be an excellently balanced heat storage material exhibiting a “water-intercalation mechanism”.

## Introduction

The increased demand for energy has strongly called for innovative developments of highly efficient solar cells, excellent high energy-density rechargeable batteries, thermal heat storage devices, etc. The significance of heat storage^1–10^ has been increasing in terms of the efficient utilization of low-grade waste heat, because about 60–70% of waste heat is discharged as the heat between 100 and 200 °C^11^. In general, heat storage materials fall into three categories, the sensible heat storage, the latent heat storage via phase-change mechanism regardless of the presence/absence of working pairs, and the thermochemical heat storage including chemical reaction. Desired functions for heat storage materials are high volumetric or gravimetric energy density, high cyclability, no pyrolysis, the feasibility of low-temperature charge to utilize low-grade waste heat, small hysteresis, fast reaction rate, fast reactant transport, heat conduction, fast water penetration in case of using water, etc.

Although lots of studies and review papers have reported many heat storage materials belonging to the above-mentioned categories^10,12–17^, there are few well-balanced materials that combine the energy density, cyclability, fast charge/discharge rate. For example, heat storage materials inevitably must possess a strong environmental resistance. When deliquescence occurs, mass transfer cannot proceed due to inactive coating by the resolvent, which causes ill cyclability. Moreover, even if the energy density is excellently high, a low reaction rate and reversibility come to be problematic issues, for example, in the cases of MgO/H_2_O (irreversible reaction)^1,4,18–21^, MgSO_4_/H_2_O (amorphization)^22,23^, CaO/H_2_O (particle fragmentation)^24^, CaCl_2_/H_2_O (e.g., liquefaction, significant degradation of mass transfer)^2,25^, etc. Especially, in MgSO_4_/H_2_O system, sluggish hydration kinetics hinders reversible charge/discharge process; sometimes amorphization is induced upon the dehydration process. Thus, the nonequilibrium phase should not appear if the system completely follows the equilibrium process^26^ but this is not the case, which means that the reversible reaction for heat storage is difficult to occur actually in ambient conditions.

Since deliquescence or liquefaction seems a disadvantageous characteristic for heat storage materials, solid-state reaction is preferred for cyclability. In this case, however, the strain effect becomes a key factor. As well as the lithium-ion batteries (LIBs) or rechargeable magnesium batteries, the stress effects cannot be ignored for the materials that can highly accommodate carrier ions^27–33^ (in this case molecules), which is well known to retard markedly the battery reaction process. This is a general common problem for the ion/molecule insertion materials. For example, according to Steiger et al.^23^, it has been shown that hydration of MgSO_4 _∙ H_2_O to form MgSO_4 _∙ 6H_2_O is a true solid-state reaction until its deliquescence, but its kinetics is strongly hindered due to the strain generation. Thus, the significant strain energy induced by the ion/molecule insertion sometimes exceeds the chemical driving force of the insertion, which retards the kinetic process.

To overcome this situation, allotropic solid-solid phase transformation under pressure (without any working pair) is shown to be useful for application to a heat storage material; a metastably frozen λ-Ti_3_O_5_ can transform to β-Ti_3_O_5_ under a pressure of 60 MPa, where the latter is the equilibrium phase in ambient conditions and the former phase can be obtained around 460 K and then the metastable λ-phase can be kept even by slow cooling^9^. The enthalpy change related to the phase transformation is about 230 MJ m^−3^, which is about 70% of the melting enthalpy (320 MJ/m^3^) of ice. The entropy change of the transformation is about 25 J K^−1^ per mol-Ti_3_O_5_, being comparable to the entropy change (22 J K^−1^ per mol-H_2_O) of ice melting. Therefore, the enthalpy change using this mechanism is close to those of phase-change materials (PCMs) using their melting phenomena. Since the heat discharge temperature is tunable by the application of an external pressure, the external field controlling phase transformation is indeed interesting and deserves to be further investigated.

Nevertheless, to obtain a much larger enthalpy change with the reversible phase transformation towards large heat storage, it is still effective to exploit a large entropy change such as the vaporization entropy change Δ*S*_vap_ of water that is about 145 J K^−1^ per mol-H_2_O^34^, and this value is found valid also for most of water sorption materials^35^. For the application feasibility of the heat storage materials, as mentioned above, the solid-state reaction should be utilized for storing thermal energy with a working pair H_2_O. To realize this, H_2_O should be inserted into the host material without structural change. In this view, Hatada et al.^1^ reported that one mole of La_2_(SO_4_)_3_ can accommodate one mole H_2_O while maintaining its original structure, by which rapid and reversible charge and discharge processes can be attained. However, it is necessary to further enlarge volumetric energy density by reducing the large molar volume required for accommodating one mole H_2_O.

It would deserve to note that fast rechargeability and good cyclability are the advantages of the water insertion system compared to others. The best insertion mechanism is believed to be “intercalation” in the narrow sense, as is well-known as the Li insertion mechanism to the layered-rocksalt CoO_2_ of LIB system. This is because fewer atoms are needed to store the carrier ions or molecules with little volume change before/after carrier insertion. Here we focus the layered structure of δ-MnO_2_ among its polymorphs^36,37^, which is assigned to space group *P*6_3_/*mmc*^38,39^. The interlayer distance of δ-type MnO_2_ (~7 Å) is larger than those of the common layered structures such as LiCoO_2_ (4.7 Å)^40^ and Li_2_MnO_3_ (4.7 Å)^41,42^. This is because the δ-MnO_2_ structure is stabilized by crystal water molecules and large cations such as K^+^ located at interlayers, which is derived from a typical synthesis route (e.g., thermal decomposition of KMnO_4_). It has been reported that the composition is *x* = 0.2–0.3, *n* = 0.5–0.9 in δ-K_*x*_MnO_2_ ∙ *n*H_2_O^38,43^ and it can allow insertion/extraction of Mg aquo-ion^44–46^. However, it is not clear to what degree thermochemical heat can be absorbed and released, at what rate water can be inserted and extracted, what temperature it can be charged at, i.e., what temperature dehydration occurs. It is also unclear how stable the δ-MnO_2_ structure is when the crystal water is lost.

In this work, we demonstrate that δ-K_0.33_MnO_2_ possessing a small molar volume (2.78 × 10^−5^ m^3^ mol^−1^) can accommodate H_2_O up to 0.83 H_2_O per K_0.33_MnO_2_ and allow the intercalation of 0.5 H_2_O reversibly around 120 °C at 5 °C min^−1^, whereas charge is feasible below 200 °C even at a very fast rate of 100 °C min^−1^. Thus, a birnessite-type MnO_2_ can be an excellently balanced heat storage material that combines a high energy density (~1000 MJ m^−3^), good reversibility with a small hysteresis of about 40 K, fast charge/discharge properties, strong environmental resistance, etc.

## Results

### Thermal analyses under dry or moisture condition

**Fig. 1:**
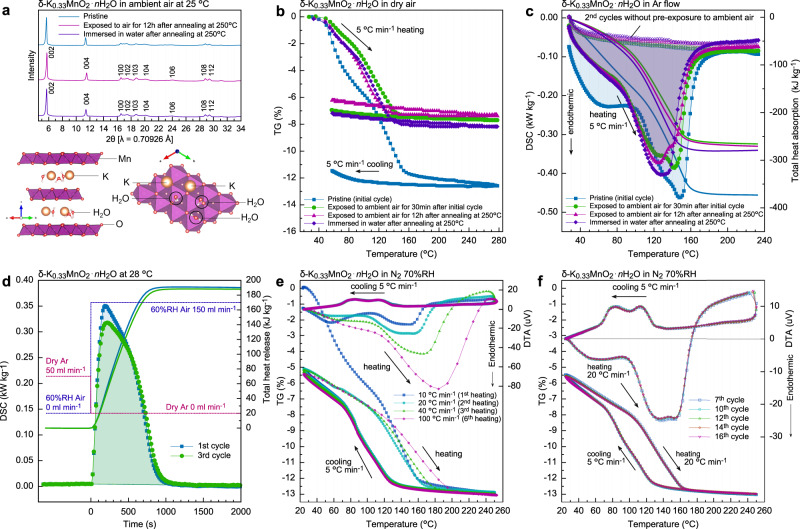
Structure and thermal properties of K_0.33_MnO_2_*n*H_2_O. **a** X-ray diffraction profiles for pristine sample, sample after annealed at 250 °C and then exposed to ambient air for 12 h, and sample after annealed at 250 °C followed by immersion into water. In the lower figure, the layered structure of δ-MnO_2_ is also shown, where the possible sites for K^+^ and H_2_O in space group #194: *P*6_3_/*mmc* are indicated in the structure. **b** TG (weight change) profile for samples of (light blue) pristine, (green) exposed to ambient air (60% relative humidity (RH) at 22 °C) for 30 min after initial cycle, (magenta, purple) exposed to ambient air for 12 h or immersed in water after annealed at 250 °C. The reference point (i.e., zero point) in TG is given with respect to the beginning of each TG measurement. **c** DSC profiles during heating (charge). The samples correspond to the respective TG measurements. **d** Isothermal DSC profiles at 28 °C while being exposed to ambient air with 60%RH moisture (discharge). **e** TG-DTA profiles in a N_2_-gas atmosphere with 70%RH moisture at 25 °C (2.2%H_2_O in N_2_ gas) at various heating rates (10–100 °C min^−1^). **f** TG-DTA cycle measurement in 2%H_2_O-N_2_ at heating 20 °C min^−1^ and at cooling 5 °C  min^−1^.

The chemical composition of the prepared sample in this study was determined to be δ-K_0.33_MnO_2_ ∙ *n*H_2_O by the inductively coupled plasma (ICP) analysis. Also from Supplementary Fig. 1, the K element is found to be homogeneously distributed in powder particles. Figure 1a shows the X-ray diffraction (XRD) profile of the pristine δ-K_0.33_MnO_2_ ∙ *n*H_2_O powder sample; the crystal structures viewed along the [110] and [001] directions are also depicted, where the possible locations of K^+^ and H_2_O are also indicated. Figure 1b shows the weight change of about 13% in thermogravimetry (TG), which was measured during heating up to 280 °C and then down to 50 °C in dry air (dew point < −70 °C). For the as-synthesized sample (blue curve in TG), the heating yields a significant weight loss, and the weight hardly recovers upon cooling under the dry condition. However, subsequently, after being exposed to ambient air with moisture (60% relative humidity (RH) at 22 °C) for 30 min (green), the weight decreases again by about 7% (from the sample sufficiently exposed to ambient air) during the second heating, and the weight is substantially unchanged upon the second cooling in dry air as well. That is, about 6% of the weight is lost from the initially synthesized state, but about 7% of weight can reversibly recover in the ambient moisture condition. The same trend in the TG profiles (magenta and purple) is observed also after being exposed to ambient air for 12 h or immersed in water after annealing at 250 °C for 5 h in air; Fig. 1a also shows that the XRD profiles of these samples are very similar to that of the pristine sample, suggesting that the frame structure is almost unchanged after annealing at 250 °C.

Figure 1c shows the heat flow of each sample measured by the differential scanning calorimetry (DSC) apparatus with a heating rate of 5 °C min^−1^. The significantly large endothermic heat is observed during the heating process up to 240 °C, and in the second cycle without exposure to ambient air, such an endothermic heat is not seen for all the samples. The endothermic heat of the pristine sample is 373 kJ per kg-pristine, and after the second cycle, the amount of heat release is 266 kJ per kg-pristine, where “kg-pristine” means the weight of the pristine state “δ-K_0.33_MnO_2_ ∙ *n*H_2_O” including crystal water. This energy release is reproducible in cycle treatments, therefore indicating a reversible reaction. Thus, the weight loss and endothermic heat are strongly suggested to be related to the deintercalation of water upon heating. It is noted here that the heat absorption of pristine δ-K_0.33_MnO_2_ ∙ *n*H_2_O (373 kJ kg^−1^) larger than those of the other samples (266–280 kJ kg^−1^) originates from the extra heat absorption at lower temperatures (detected as a shoulder in the DSC profile between 40 and 80 °C), suggesting that the excess water was confined in pristine δ-K_0.33_MnO_2_ ∙ *n*H_2_O during the synthesis process.

Figure 1d shows an isothermal heat release profile measured at 28 °C for dried δ-K_0.33_MnO_2_. Pristine δ-K_0.33_MnO_2_ ∙ *n*H_2_O was dried by heating up to 240 °C under an Ar atmosphere (charge) and then exposed to ambient 60%RH air at 28 °C (discharge); the exposure to humid air rapidly caused an exothermic reaction of dried δ-K_0.33_MnO_2_. The exothermic heat amounts to 190 and 188 kJ kg^−1^ in the first and third cycles, respectively, within about 1000 s, suggesting the high reversibility and reasonable rate capability of heat release.

To make sure whether the water intercalation indeed occurs in the ordinary time scale, we have performed the TG-DTA (differential thermal analysis) measurements in a humidity of 70%RH at 25 °C (atmosphere of 2.2% H_2_O-N_2_ gas) flow at various heating rates from 10 to 100 °C min^−1^ and fixed cooling rate of 5 °C min^−1^ by using Thermo plus EVO2 TG-DTA8122/HUM-1 (Rigaku). As shown in Fig. 1e, under this moisture atmosphere condition, the weight loss is completely compensated upon cooling below 120 °C and finally revert to the original weight on and after the second cycle. Moreover, the DTA profile (temperature difference) corresponds to the TG profile in each cycle; the sample temperature decreases when the sample weight decreases and vice versa. This indicates that the endothermic reaction occurs when the H_2_O molecules absorbed in the crystal are liberated, and the exothermic reaction appears when water is inserted into the crystal below 120 °C. Thus, a series of the reactions are on hydration and dehydration process from the viewpoint of the TG-DTA analysis. Given that a state of about 13% loss in weight around 260 °C can be regarded as just K_0.33_MnO_2_ (i.e., *n* = 0), the pristine state is *n* = 0.83 in δ-K_0.33_MnO_2_ ∙ *n*H_2_O (114.84 g mol^−1^), and the hydrated state before the weight loss of about 7.8% in the second cycle corresponds to *n* = 0.50 (108.76 g mol^−1^). Consequently, the stored energy is 42.9 kJ per mol-K_0.33_MnO_2_ for *n* = 0.83, and 30.5 kJ per mol-K_0.33_MnO_2_ for *n* = 0.50, which means that 51.9 kJ per mol-H_2_O (derived from the former) and 61.7 kJ per mol-H_2_O (from the latter). The released heat shown in Fig. 1d amounts to 21.82 kJ per mol-K_0.33_MnO_2_ for *n* = 0.50, corresponding to 44.07 kJ per mol-H_2_O. These differences are discussed later.

Furthermore, as to the kinetics of hydration and dehydration, even when heating at 100 °C min^−1^, the dehydration completes around 190–200 °C, which is slightly higher than the relevant temperature (160 °C) at a heating rate of 10 °C min^−1^. Thus, this small difference of only 30 °C in the dehydration-completion temperature means that the H_2_O diffusion in the crystal is quite rapid. Figure 1f shows completely the same TG-DTA profile of each cycle, indicating very good reversibility of the hydration/dehydration cycle. Note that the slope of TG upon cooling below 120 °C and that upon heating between 100 and 160 °C are substantially the same. Also as seen in Fig. 1e, although the TG profiles upon heating at 10 and 20 °C min^−1^ are quite different in the low-temperature range because the initial amount of water is different, they are substantially overlapped above 150 °C. Namely, the same profile as that at 20 °C min^−1^ is expected to be obtained reasonably also by heating at 5 °C min^−1^. These indicate that the heating and cooling rate between 5 and 20 °C min^−1^ substantially would yield little difference in the hydration and dehydration kinetics.

### Structure change under dry or moisture condition

**Fig. 2:**
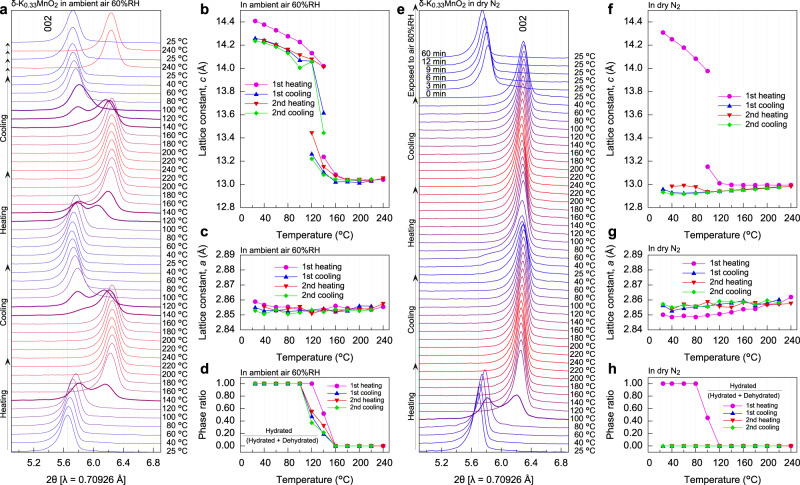
Structure changes accompanied by dehydration and hydration cycles. **a** In situ X-ray diffraction (XRD) profiles for δ-K_0.33_MnO_2_ in an ambient atmosphere (60%RH at 24 °C) for thermal cycle processes between 25 and 240 °C. **b**,**c** Lattice constants *c* (**b**), and *a* (**c**) determined from the XRD profiles. **d** Fraction of hydrated phase obtained from the XRD intensities of the hydrated and dehydrated phases. **e**–**h** In situ X-ray diffraction profiles (**e**), the lattice constants *c* (**f**), and *a* (**g**), and the phase fraction (**h**) measured under a dry N_2_-gas flow without moisture. In (**e**), the sample was exposed to an ambient air of 80%RH at 24 °C.

Actually, how much different is the dehydrated structure from the original structure? Fig. 2a shows the 002 diffraction peak profiles upon heating and cooling processes in ambient conditions (60–80%RH at about 20–25 °C) or under a dry N_2_ gas flow; the 002 diffraction is a characteristic one of δ-K_0.33_MnO_2_ representing the interlayer distance along the *c* axis. As shown in Fig. 2b, the lattice constant *c* of the pristine sample is 14.4 Å, which gradually decreases down to 13.0 Å with dehydration by an increase in temperature, and it increases with hydration by a decrease in temperature. The slightly larger lattice constant *c* observed in the first heating can be understood from the fact that pristine δ-K_0.33_MnO_2_ ∙ *n*H_2_O (*n* = 0.83) contains excess water while *n* basically ranges from 0.5 to 0.0 after the second cycle. In contrast, as seen in Fig. 2c, the lattice constant *a* determined from the XRD profile in a wide two-theta range (Supplementary Fig. 2) is almost unchanged during heating and cooling. Moreover, as shown in Fig. 2a, b, upon heating the peak split occurs around 140 °C, indicating a two-phase region, and then an almost dehydrated phase appears around 160 °C. Although upon cooling the two phases appear again around 140 °C as well as upon heating (meaning little hysteresis), the high-temperature phase can be retained down to 120 °C. Figure 2d summarizes the fraction of hydrated phase estimated from the XRD intensities, indicating the two-phase region between 120 and 140 °C. Thus, such a small temperature hysteresis would be due to the almost equilibrium condition during the in situ high-temperature XRD measurements (spent for about 30 min at each temperature). Further interestingly, it deserves to note that the lattice constant *c* tends to be influenced by the phase fraction. For example, as seen in Fig. 2b, d, when the fraction of hydrated phase is about 0.6 in the second heating process (the red plot at 120 °C) the dehydrated phase is significantly strained, whereas the hydrated phase is strained when the fraction is as small as about 0.2 in the second cooling process (see the green plot at 140 °C). This would be due to the coherent strain effect that is yielded when the layered structure is maintained but the crystal water is included inhomogeneously.

On the other hand, in the condition of a dry N_2_ gas flow, as shown in Fig. 2e–h, the first dehydration process causes a decrease in the interlayer distance, but the 002-diffraction peak is substantially unchanged after dehydration even regardless of temperature increase and decrease; see also Supplementary Fig. 3 for a wide two-theta scan. After being exposed to the ambient air (80%RH at 24 °C), the interlayer distance reverts closely to the original value, although it does not completely. Namely, this shrinkage of the *c* axis (or interlayer distance) is found to be caused by the deintercalation of crystal water to evaporate.

From the above XRD measurements, the lattice constants and mass density of the pristine state and reversibly hydrated state (the initial in the second cycle) can be determined. Since the mass densities of δ-K_0.33_MnO_2_ ∙ 0.83H_2_O and δ-K_0.33_MnO_2_ ∙ 0.5H_2_O are, respectively, determined to be 3740 kg m^−3^ and 3588 kg m^−3^, the maximum heat storage amounts to 1395 MJ m^−3^ and the thermal energy reversibly stored is 1007 MJ m^−3^. These energy densities of δ-K_0.33_MnO_2_ are indeed excellent among those of commonly known PCMs, which is discussed in the “Discussion” section.

### Water-absorption energy estimated by ab initio calculation

**Fig. 3:**
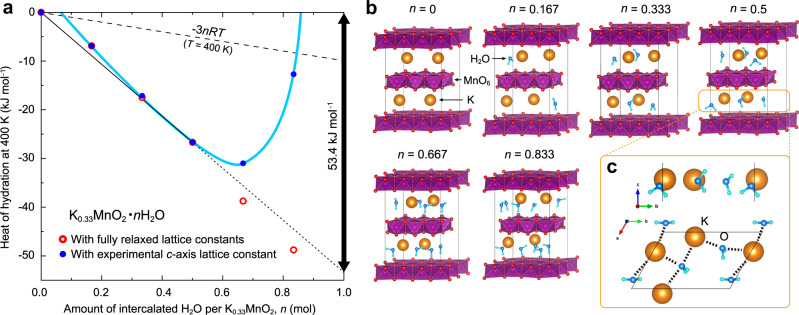
Ab initio calculations of hydration heat and optimized structure of δ-K_0.33_MnO_2_ ∙ *n*H_2_O. **a** Heat of hydration (enthalpy benefit due to the water intercalation) obtained by ab initio calculation for δ-K_0.33_MnO_2_ ⋅ *n*H_2_O, **b** the optimized structure with H_2_O molecules for each content of water. **c** A typical coordination of K^+^ ions and H_2_O molecules sited in an interlayer for *n* = 0.5, where the averaged distances of K-O(H_2_O) and K-H are 2.83 Å and 2.97 Å, respectively. The internal energy of H_2_O gas (=3*nRT*) is considered by assuming that H_2_O is an ideal gas with degree of freedom 3 (translational) + 3 (rotation). The two sets, free relaxed (red) and fixed lattice constants (blue), are calculated.

In order to verify the validity of the hydration energy obtained experimentally, with the aid of the ab initio calculation (VASP package), we have computed the internal energy at zero Kelvin of the δ-K_0.33_MnO_2_ lattice with *n* mol of H_2_O molecules, *E*(*n, T*) at *T* = 0, for a supercell δ-K_4_Mn_12_O_24_ ∙ 12*n*H_2_O. Taking account of the molar formation energy ε of H_2_O molecule and kinetic energy (=3*RT*) of 1-mol water vapor, the heat of hydration Δ*H*_hyd_(*n*, *T*) is given by Δ*H*_hyd_(*n*, *T*) ≡ *E*(*n*, *T*) – [*E*(0, *T*) + *n*ε + 3*nRT*]. The values obtained for *T* = 400 K (127 °C) are plotted in Fig. 3a, where it is assumed that *E*(*n*, *T*) – *E*(0, *T*) – *n*ε = *E*(*n*, 0) – *E*(0, 0) – *n*ε, and the latter of the equation can be computed. This is considered reasonable as a first approximation. In the fully relaxed condition (red), the hydration energy decreases monotonically with an increase in *n*, but the lattice constant of the *c* axis comes to be much larger than the experimental one (Supplementary Fig. 4). Then, we have imposed a constraint condition that the lattice constants are fixed so as to equal the experimental values to the calculations. In such a condition (blue), the hydration energy shows a downward convexity like a blue curve. This can explain well the experimental phenomenon that the pristine state has a larger crystal water inside the host structure (*n* = 0.83) but only the half quantity (*n* = 0.5) can be recovered in the next hydration process. Namely, it would be reasonable to consider that the initial hydrated state can be attained only by the synthesis process.

In the calculated *n*-dependence of hydration energy, there is a slight energy hump at *n* = 0.167, which means that the two-phase region should appear in a low concentration range of water (i.e., at small *n*). Since the TG profile in Fig. 1e has a typical feature of the single-phase reaction, it is difficult to judge what weight in TG the two-phase region appears at. In the light of the XRD results in Fig. 2b, d, since it appears around 140 °C upon heating, which indicates that the two phases coexist in the vicinity of the end of dehydration process upon heating. Therefore, it is concluded that this ab initio calculation can reproduce well the trend of the present experimental result. What is the most important here is that the heat of hydration Δ*H*_hyd_ at *T* = 400 K is estimated to be 53.4 kJ per mol-H_2_O from the common tangent of the points at *n* = 0 and at *n* = 0.5, which is in fairly good coincidence with the DSC data (61.7 kJ per mol-H_2_O) obtained for δ-K_0.33_MnO_2_ ∙ 0.5H_2_O; see Fig. 1c and the relevant description.

The optimized structures under the given constraint condition are shown in Fig. 3b, c. The initial sites of K^+^ ions in the completely dehydrated structure are 2*b* and in-between sites of 2*b* and 2*c* in space group #194 (*P*6_3_/*mmc*); see also Supplementary Fig. 5. In contrast, when *n* = 0.167 or more than the value, K^+^ ions and H_2_O molecules are no longer located at the 2*b* and 2*c* sites, being consistent with the previous study on the structure analysis of the δ-(K)MnO_2_ with H_2_O molecules^38^. The inserted water molecules coordinate to the K^+^ ions and pull them out of their stable positions in the crystal, resulting in a random structure that would realize the transverse isotropy in the *ab* plane. The trend that the O atom of H_2_O molecule is bound to K^+^ ion (Fig. 3c) is in good agreement with the previous report by Chen et al.^47^. Although K^+^ ions are randomly distributed (in the present calculation), the attractive interaction between K^+^ ions and H_2_O molecules would result in the ice-like (or solid-like) rather than liquid-like in the state of interplane H_2_O molecules, which is suggested from the entropy analysis in the “Discussion” section. This is consistent with the view expected from Raman spectroscopy measurements (Supplementary Fig. 6). The Raman spectra of δ-K_0.33_MnO_2_ show a new peak at 560 cm^−1^ in the hydrated states, whereas the Raman spectrum of δ-K_0.06_MnO_2_ (after leaching K^+^ ions) hardly shows such a peak even by hydration. This suggests that the insertion of water induces a new vibration mode associated with the hydration structure of K^+^. This is also inferred from the orientation of the oxygen atoms of the water molecules toward the K^+^ ions, as can be seen in Fig. 3c. In short, a physical picture of the interlayer dissolution of K^+^ ions can be drawn. In addition, it is also shown that δ-K_0.06_MnO_2_ with few K ions is unstable even above 150 °C and dehydration energy is less than that in δ-K_0.33_MnO_2_ (see also Supplementary Fig. 7), suggesting that the interlayered K^+^ ions not only play a role in strongly stabilizing this layered structure but also contribute to the incorporation of H_2_O into the stabilized crystal as a phenomenon of interlayer dissolution of K^+^ ions.

## Discussion

**Fig. 4:**
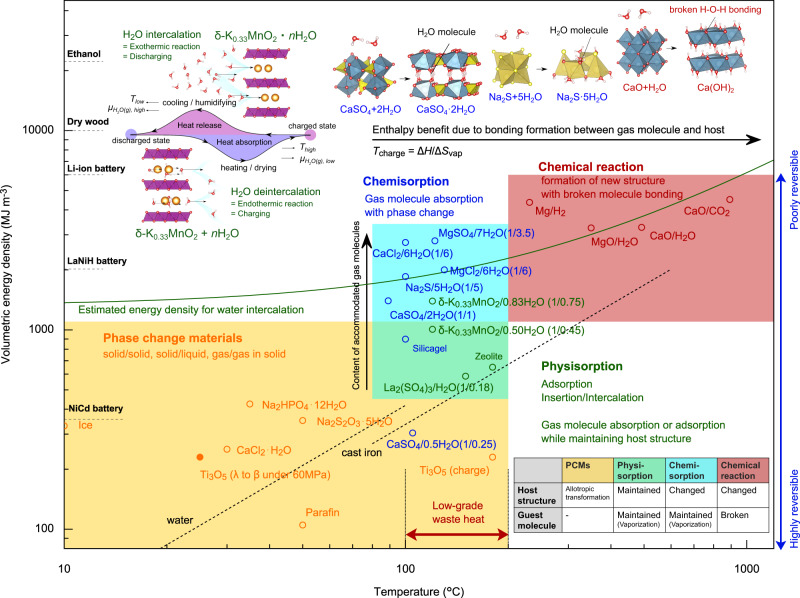
Volumetric energy density versus temperature plots for various types of heat storage materials. The data other than the present data are taken from the references^1–9^. The heat storage materials represented by blue and red change the initial host structure when accommodating gas molecules, where gas molecules are kept inside heat storage materials in the blue category but the bonding of molecules are broken inside heat storage materials in the red category. In contrast, heat storage materials denoted by green can absorb gas molecules while maintaining the host structure. The figure in parentheses denotes the ratio of “number of atoms in guest” to “number of atoms in host”. The dashed lines are depicted for water (heat capacity 4.18 kJ K^−1^ kg^−1^, density 1000 kg m^−3^) and cast iron (0.46 kJ K^−1^ kg^−1^, 7280 kg m^−3^) given that the reference temperature is set to 0 °C. The green curve indicates the ideal maximum energy density per dehydrated substance volume (based on the manner presented here) that is estimated for water-intercalation materials.

Based on the previous reports^1–9^ and the present results, Fig. 4 summarizes the volumetric energy density versus temperature plots for various types of heat storage materials. In this figure, the heat storage materials are classified into “sensible heat storage” (dashed line), “phase change materials (PCMs)” (denoted by yellow), “sorption materials” (green and blue), and “chemical reaction” (red). The “phase change materials” usually utilize the allotropic phase transformation (e.g., melting). In “sorption”, there are two types of materials, physisorption (green) and chemisorption (blue), and gas molecules themselves are basically unchanged in the absorption solid. The former maintains the host structure when absorbing gas molecules, whereas the latter causes the structure to change with gas absorption. In “chemical reaction”, the bonding form of gas molecules are broken to be accommodated inside the solid substance while changing its original structure. These features are summarized in the inset table.

Gas-molecule insertion, intercalation, or surface-adsorption materials belong to “physisorption”. Basically, insertion or intercalation materials can absorb gas molecules inside while maintaining the host structure and only utilize the phase transformation between gas and firmly absorbed state in solid (i.e., vaporization); in this sense, the physisorption materials also belong to PCMs. In the first place, in order to store a large thermal energy, it is quite essential to consider how to freeze such a high entropy state down to the operating temperature. In other words, the intercalation-type PCMs using water as a working pair enable effectively to utilize the high entropy state of water vapor by freezing dry of their dehydrated state of PCMs.

Here let us delve into the intercalation materials, such as δ-MnO_2_, that exploit the “water vaporization”. Under constant pressure, since the heat of hydration (enthalpy benefit) is given by Δ*H*_hyd_ = *T*Δ*S*_hyd_, in the case of the present ab initio calculation, given that the reaction temperature is 400 K, the hydration entropy Δ*S*_hyd_ is inversely evaluated to be 134 J K^−1^ per mol-H_2_O. It deserves to note that the hydration entropy Δ*S*_hyd_ is very close to the vaporization entropy Δ*S*_vap_ from a solid state (~145 J K^−1^ per mol-H_2_O)^35^. Actually, in the case of the present experiment (61.7 kJ per mol-H_2_O), the (de)hydration temperature is estimated to be 425 K (152 °C), which is very close to the experimental value (120–160 °C), as seen in Figs. 1e and 2b. In addition, the isothermal water-intercalation DSC profile (Fig. 1d) allows precise estimation of entropy change. The exothermic heat of 44.07 kJ per mol-H_2_O observed at 28 °C (301 K) indicates the entropy change of 146.4 J K^−1^ per mol-H_2_O. The good agreement of this entropy change accompanied by water intercalation with the sublimation entropy of water (~145 J K^−1^·per mol-H_2_O) suggests that the water molecules in the interlayer space of δ-MnO_2_ is ice-like (or solid-like) rather than liquid-like, and this interpretation is in accordance with the previous report by Chen et al.^47^. Thus, the hydration energy (i.e., amount of heat storage with H_2_O as a working pair) is dominated by the vaporization entropy of H_2_O and H_2_O-release temperature. This would be true as far as H_2_O gas is separated out from the absorption substance in the dehydrated state, even if the H_2_O molecules are broken, for example, changed to H/OH inside the substance.

Based on the above argument, consequently, the heat storage due to the enthalpy gain is given by Δ*H* = *T*_charge_Δ*S*_vap_ per one mole of water, where Δ*H* ≥ 54 kJ per mol-H_2_O is guaranteed if the charge temperature *T*_charge_ ≥ 373 K. Therefore, although the operating temperature of heat storage materials may frequently be room temperature, it would be preferred that the charge temperature *T*_charge_ (i.e., H_2_O-release temperature) is as high as possible in terms of a large capacity of thermal energy storage. Nevertheless, from the practical point of view, a possible charge temperature is in the range of low-grade waste heat (373 K ~ 473 K)^11^. Thus, the upper limit of the heat storage can be estimated to be Δ*H* ~69 kJ per mol-H_2_O (at 473 K) for the water-intercalation-type heat storage materials. If larger heat storage materials are demanded, we have to choose “chemisorption” or “chemical reaction” materials. However, since both heat storage materials cannot absorb gas molecules while maintaining their host structure, the reversibility comes to worse in general. Especially, in “chemical reaction” materials, the charge temperature *T*_charge_ (=Δ*H*/Δ*S*_vap_) inevitably becomes higher (over the low-grade waste heat level) due to the compensation of large Δ*H*.

It is necessary for increasing the volumetric heat-storage capacity to reduce the molar volume or number of atoms required for accommodating one molecule of H_2_O. Therefore, what we have to discuss for intercalation materials is how much volume of the host material is required for the H_2_O accommodation. Focusing on the difference between chemisorption and physisorption materials, let us infer the volume needed for hosting one mole H_2_O without any structure change. As suggested by blue-circle data in Fig. 4, it would be reasonable to consider that the upper limit of the ratio of “number of atoms in guest” to “number of atoms in host” would be 1/1 (the figure in the parentheses); for example, CaSO_4_/2H_2_O (1/1), as well as Na_2_S/5H_2_O (1/5) with the guest-host atom number ratio of unity or higher, cannot maintain the host structure with H_2_O absorption as depicted in the inset. Thus, under the constraint case where H_2_O can be stored without any change of the host structure, the maximum amount of H_2_O accommodation would be empirically 1/1 in the atom-number ratio like MnO_2_/H_2_O.

Here, for the sake of simplicity and versatility, the volume of one mole of atoms is supposed to be 1 × 10^−5^ m^3^ per mol-atom; see the relevant description in Methods. Then, the volume of the host matrix required for storing one mole of H_2_O is 3 × 10^−5^ m^3^ per mol-3atoms. Based on this argument, the ideal stored energy density for water-intercalation materials is given by Δ*H* = (3 × 10^−5^)^−1^*T*Δ*S*_vap_ = 4.833 *T* MJ m^−3^, which is also drawn by the green curve in Fig. 4. Consequently, this gives that the stored energy around 400–473 K is about 1900–2290 MJ m^−3^, which is around an ideally maximum amount of stored enthalpy by using vaporization of water with low-grade-waste heat. Note that the volumetric energy density obtained by this equation corresponds to one based on the unit volume before hydration and actually the nominal value shifts downward. Thus, it is noteworthy to mention that we obtained a very excellent and rationalized value exceeding 1000 MJ m^−3^ of the stored heat with 0.5 mol-H_2_O per 1-mol δ-(K)MnO_2_ in the present discovery. Incidentally, even if H_2_ gas or CO_2_ (other than H_2_O gas) is a counterpart of working pair, the above idea is substantially applicable because Δ*S*_vap_ is almost the same (140–145 J K^−1^ per mol-gas) for various gases at the temperature where the standard Gibbs formation energy Δ*G*^o^ = 0 holds; for example, Δ*S*_vap_ = 142 J K^−1^ per mol-H_2_ at 300 °C for Mg + H_2_ = MgH_2_ and Δ*S*_vap_ = 143 J K^−1^ per mol-CO_2_ at 900 °C for CaO + CO_2_ = CaCO_3_^34^. Why this green curve has deviated toward the lower side in the case of MgH_2_ is that H atom is much smaller than the other atoms.

The significance of the present discovery is the demonstration that the mechanism of “water intercalation” in heat storage materials is very advantageous and favorable in terms of their reversibility and reaction rate and, especially, in terms of the best heat storage capacity among PCMs. Furthermore, the layered-structured (K)MnO_2_ crystal possesses lots of superiorities for the heat storage materials, in that it is an oxide (not sulfate, sulfide, chloride) that would have the strongest environmental resistance. That is, of course, this material cannot be degraded by environmental oxygen, and can be immersed in water to dissipate heat since it is not dissolved. For the application of heat storage materials, it is crucial to somehow attain a supercooled high entropy state, and freezing dry of δ-K_0.33_MnO_2_ after dehydration thermodynamically corresponds to freezing of the water-vapor state that possesses a quite high entropy. Thus, the use of an excellently balanced δ-K_0.33_MnO_2_ with moisture/water in an open system would benefit us towards the application of potential heat storage materials exploiting low-grade waste heat.

## Methods

### Materials preparation and characterization

δ-MnO_2_ was prepared following Gaillot et al.^38,39^ KMnO_4_ (Wako Chemical) crystalline powder was thermally decomposed at 700 °C in air. In order to remove soluble by-products, the powder was washed with distilled water several times until a colorless solution was obtained. The remaining insoluble black powder was filtered and dried in vacuum at 80 °C. ICP optical emission spectrometry confirmed that the composition of the resulting powder was K_0.33_MnO_2_.

X-ray powder diffraction (XRD) analysis was performed using a Rigaku SmartLab diffractometer with MoKα radiation (*λ* = 0.70926 Å). In situ·XRD analysis at controlled temperatures was carried out at a heating rate of 5 °C min^−1^ with periods of constant temperature for 30 min to collect diffraction patterns. The samples were placed in ambient air atmosphere (60%RH at 24 °C) or dry N_2_ atmosphere during measurement. Crystal structures of MnO_2_ polymorphs were visualized with the VESTA program^48^.

For reference, transmission electron microscopy (TEM) observation was also conducted by JEM-2000EXII (JEOL) for as-prepared sample (initially in a sufficiently hydrated state), the results of which are given in Supplementary Fig. 8.

### Thermal analyses under dry or moisture condition

DSC was carried out using NETZSCH DSC 404 F3 Pegasus for c.a. 10 mg of MnO_2_ at a heating rate of 5 °C min^−1^ under Ar flow at a rate of 50 ml min^−1^.

Preliminary thermogravimetric analysis under dry air (dew point < −70 °C) was carried out using SHIMADZU DTG-60/60H at a heating rate of 5 °C  min^−1^. Thermogravimetric analysis under controlled humidified N_2_ atmosphere (70%RH) was performed using Thermo plus EVO2 TG-DTA8122/HUM-1 (Rigaku). The rate capability was measured for the first six cycles at a heating rate of 10, 20, 40, 60, 80, and 100 °C min^−1^ whereas the cooling rate was fixed at 5 K min^−1^. Cyclability was subsequently assessed up to 16th cycles at a heating rate of 20 °C min^−1^ and a cooling rate of 5 °C min^−1^.

### Ab initio calculation

The ab initio calculations based on the density functional theory were performed by using the Vienna ab initio simulation package (VASP) code^49,50^ with the generalized gradient approximation of Perdew-Burke-Ernzerhof (GGA-PBE) to treat the exchange-correlation functional^51^. A plane wave energy cut-off of 520 eV was used. For structural relaxation and total energy calculations, on-site Coulomb interactions were corrected with the Hubbard *U* parameter of 3.9 eV^52,53^. In order to reproduce the composition of δ-K_0.33_MnO_2_ with partial occupancy at the K sites, a 2*a*_1_ × 3*a*_2_ × *c* supercell of K_4_Mn_12_O_24_ was constructed based on the hexagonal δ-Mn_2_O_4_ unit cell, followed by appending four K atoms (initially at arbitrary 4 sites of 12 sites in 2*c*) and an appropriate number of H_2_O molecules (12*n*) in between the MnO_2_ slabs. External and internal relaxations were performed under the angle constraint (*α* = *β* = 90°, *γ* = 120°) until the energy and forces converged to 10^−6^ eV and 5 × 10^−3 ^eV nm^−1^, respectively. For the total energy calculation of H_2_O, a hexagonal supercell with *a* = *c* = 15 Å including one H_2_O molecule was constructed and internal relaxation was performed.

### An estimation of typical atomic volume

A typical volume of one mole atoms without regard to the atomic species was determined as follows. For example, in the case of Al metal, the molar volume is about 1 × 10^−5^ m^3^ per mol-Al. Moreover, in the case of δ-K_0.33_MnO_2_ (99.84 g mol^−1^, 3597 kg m^−3^), the molar volume is 2.78 × 10^−5^ m^3^ per mol-δ-K_0.33_MnO_2_, so that dividing by 3.33 atoms gives 0.833×10^−5^ m^3^ per mol-atom, and in the case of water, the reciprocal of 55.6 mol-H_2_O L^−1^ (=167 × 10^3^ mol-atom m^−3^) gives 0.6 × 10^−5^ m^3^ per mol-atom. Although the atomic radii of water, metals, and oxides are different, the molar volume of each element is still roughly approximated to be 1 × 10^−5^ m^3^ per mol-atom, ranging between 0.6 × 10^−5^ and 1 × 10^−5^ m^3^ per mol-atom in the examples.

## Supplementary information

Supplementary Information

## Data Availability

All the data that support the findings of this study are available within the article and Supplementary Information file, or from the corresponding authors upon request.
