# Supplementary material for: Excellently balanced water-intercalation-type heat-storage oxide
Source: Nat Commun. 2022 Mar 17;13:1452. doi: 10.1038/s41467-022-28988-0 (PMC8931080; doi:10.1038/s41467-022-28988-0)
Supplement: Supplementary file 1 — Supplementary Information [file 41467_2022_28988_MOESM1_ESM.pdf]

Supplementary Information

Excellently balanced water-intercalation-type heat-storage oxide

Takuya Hatakeyama *et al*.

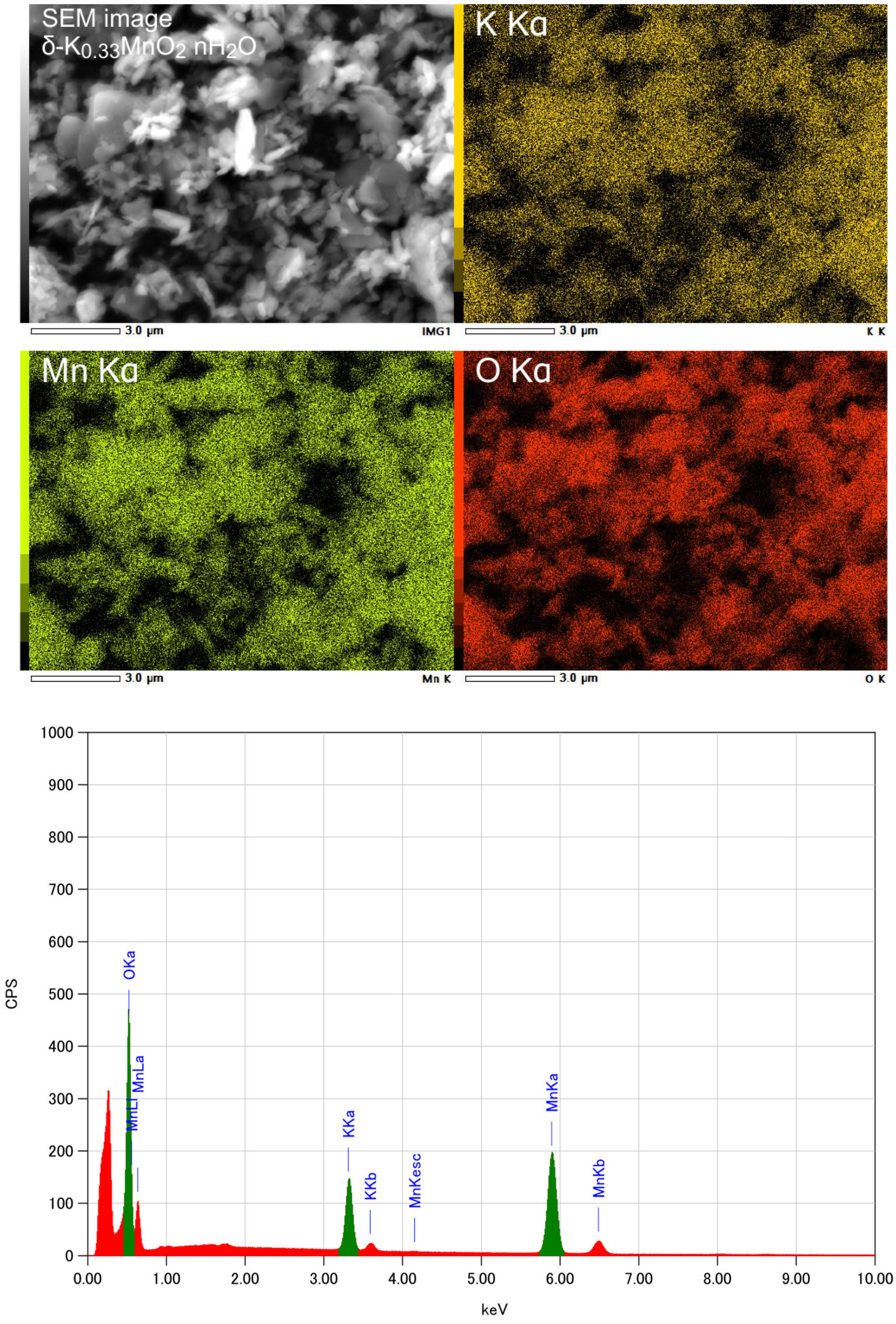

Supplementary Figure 1. Scanning electron microscopy (SEM) image and chemical element mapping images of the pristine δ-$K_{0.33}MnO_2 \cdot nH_2O$ powder measured using energy-dispersive X-ray (EDX) spectroscopy under an accelerating voltage of 15 kV. Yellow, green and red colors represent K, Mn and O, respectively. From the EDX spectrum, the K/Mn ratio is estimated to be about 0.28, being slightly smaller than the evaluation from the ICP analysis but comparable.

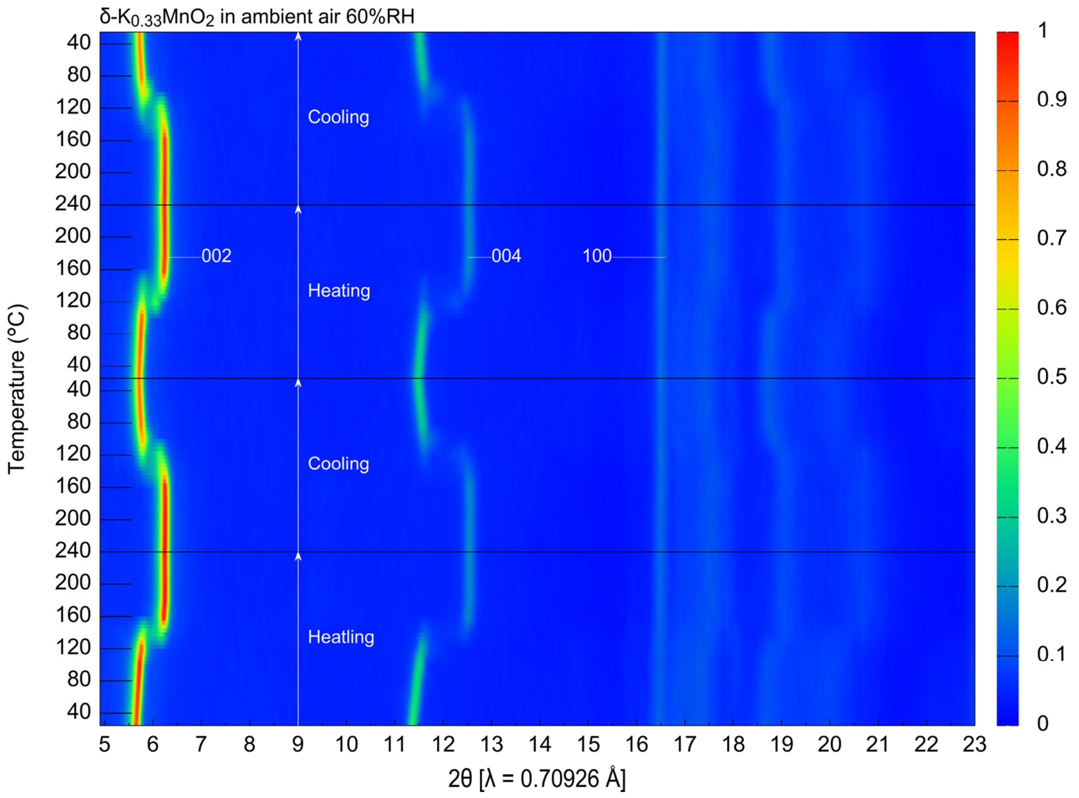

Supplementary Figure 2. In-situ X-ray diffraction profiles of δ-$K_{0.33}MnO_2$ in an ambient atmosphere (60%RH at 24°C) during thermal cycle processes between 25 °C and 240 °C. X-ray diffraction profiles were collected for 30 min at every 20 °C temperature step. Temperature was controlled at a heating rate and cooling rate of 5 °C min^{-1}.

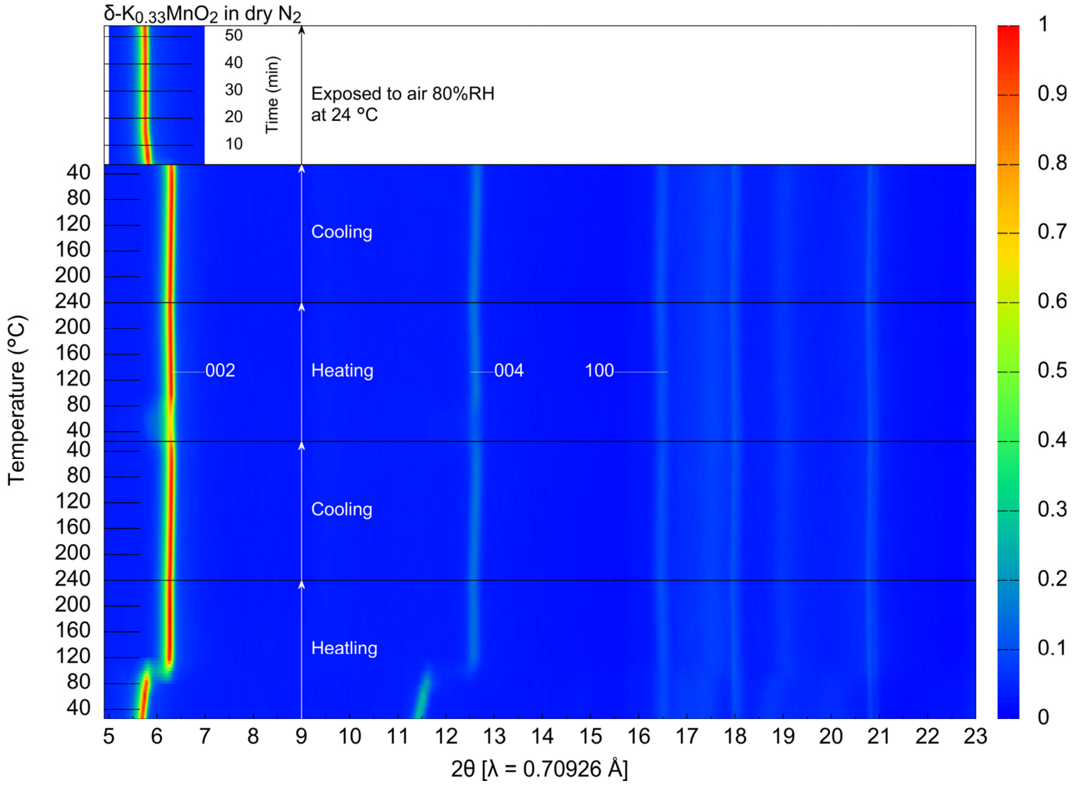

Supplementary Figure 3. In-situ X-ray diffraction profiles for δ-$K_{0.33}MnO_2$ under a dry N_2-gas flow without moisture during thermal cycle processes between 25 °C and 240 °C. After the two heating and cooling cycles, the sample was exposed to ambient air (80%RH at 24°C). X-ray diffraction profiles were collected for 30 min at every 20 °C temperature step. Temperature was controlled at a heating rate and cooling rate of 5 °C min^{-1}.

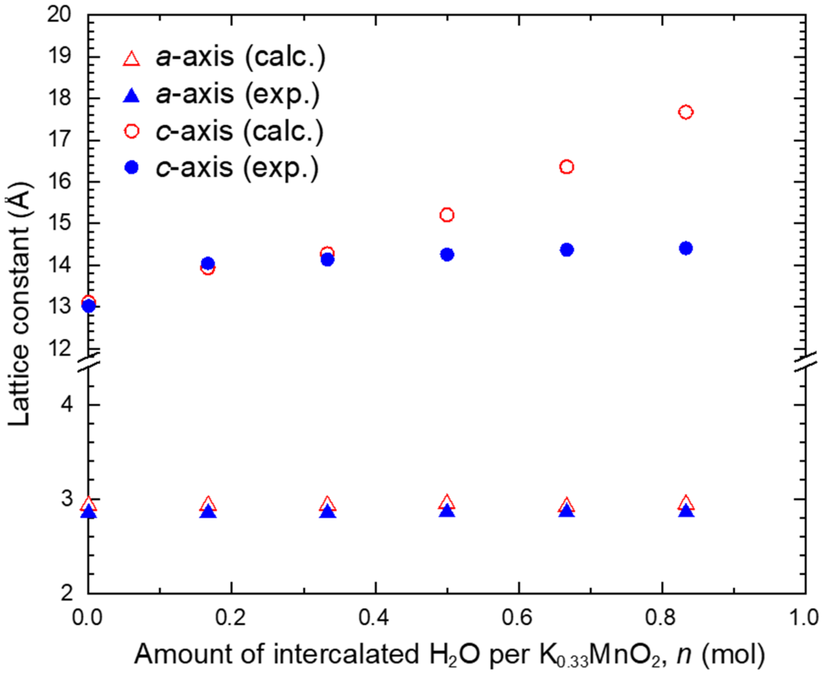

Supplementary Figure 4. Lattice constants of δ-$K_{0.33}MnO_2 \cdot nH_2O$ obtained experimentally by in-situ X-ray diffraction (solid blue) and those obtained theoretically by *ab initio* calculations in the fully relaxed condition (open red).

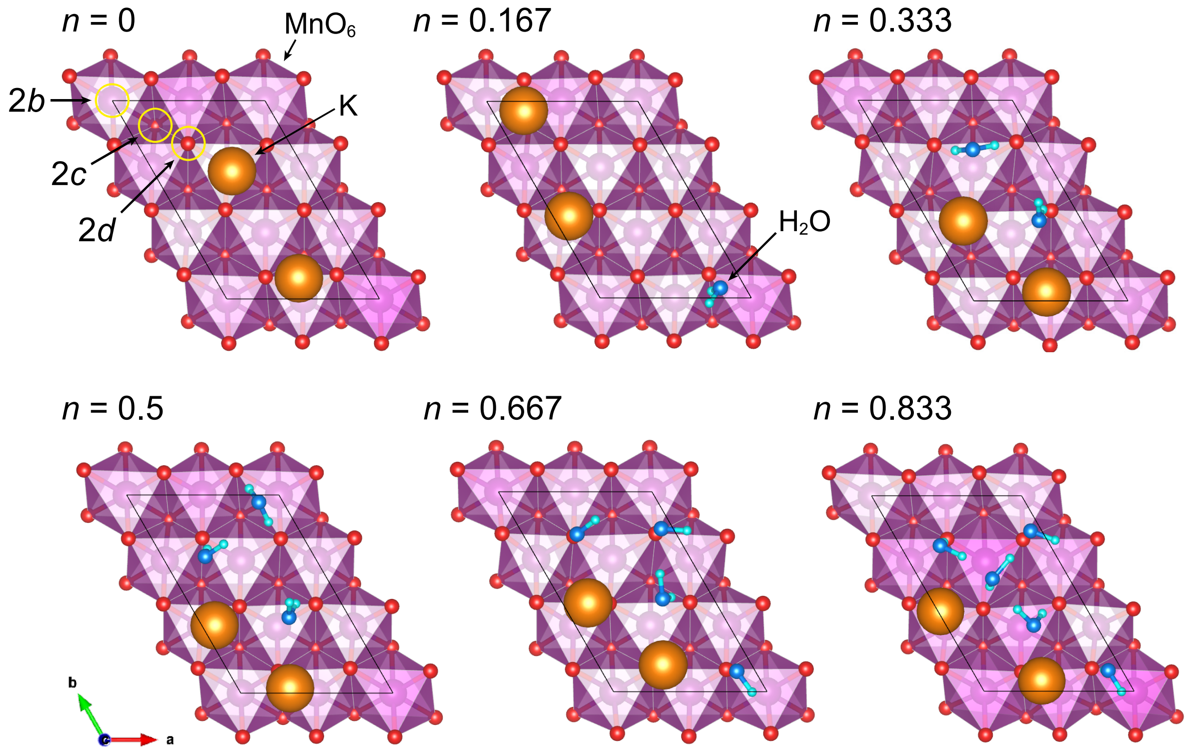

Supplementary Figure 5. Typical in-plane structures obtained after *ab initio* calculations for the δ-type $K_4Mn_{12}O_{24} \cdot 12nH_2O$ supercell for various water contents. The 2*b*, 2*c*, 2*d* sites are possible for K^+ ions and H_2O molecules for retaining the symmetry of *P*6_3/*mmc*. However, they are not located in the ideal positions in *P*6_3/*mmc*.

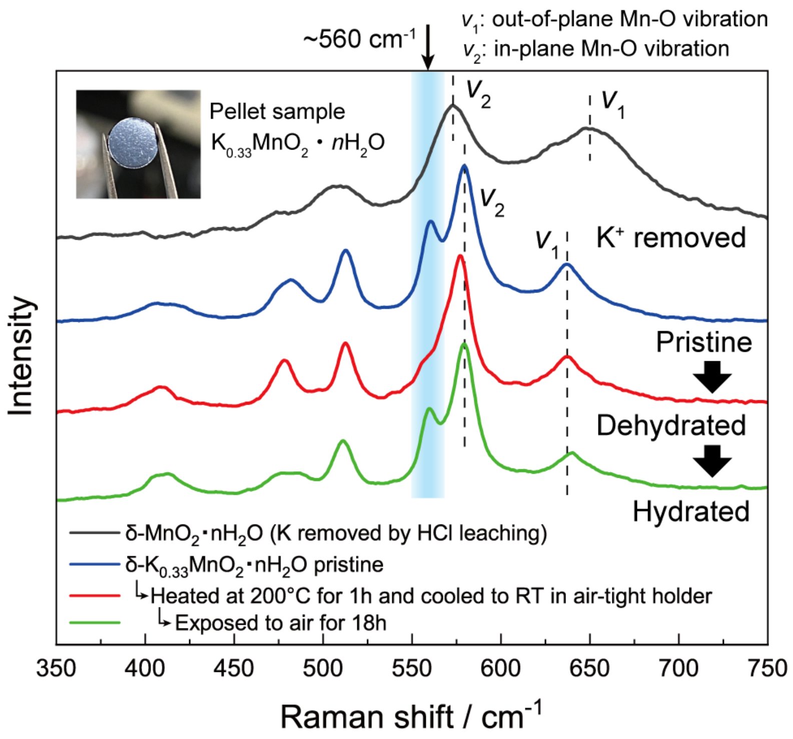

Supplementary Figure 6. Raman spectra measured for a pellet-shaped $K_{0.06}MnO_2$ made by the cold press using powder obtained by leaching K in an HCl solution (black), the pristine sample $K_{0.33}MnO_2{\cdot}0.83H_2O$ (blue), dehydrated sample $K_{0.33}MnO_2$ (red), and again hydrated sample (green). The Raman spectra in the range of 350 ~ 700 cm^{-1} can be assigned to the vibration modes of δ-MnO_2.[1,2] According to the previous studies[3-5], The v_1 mode around 620−650 cm^{-1} is attributed to the Mn-O vibration normal to the MnO_2 layer (out-of-plane Mn-O vibration), and the v_2 mode around 570−590 cm^{-1} is corresponding to the Mn-O vibration stretching along the MnO_2 layer (in-plane Mn-O vibration). Note that appearance of the peak around 560 cm^{-1} depends on the presence/absence of crystal water in MnO_2 including K^+ ions.

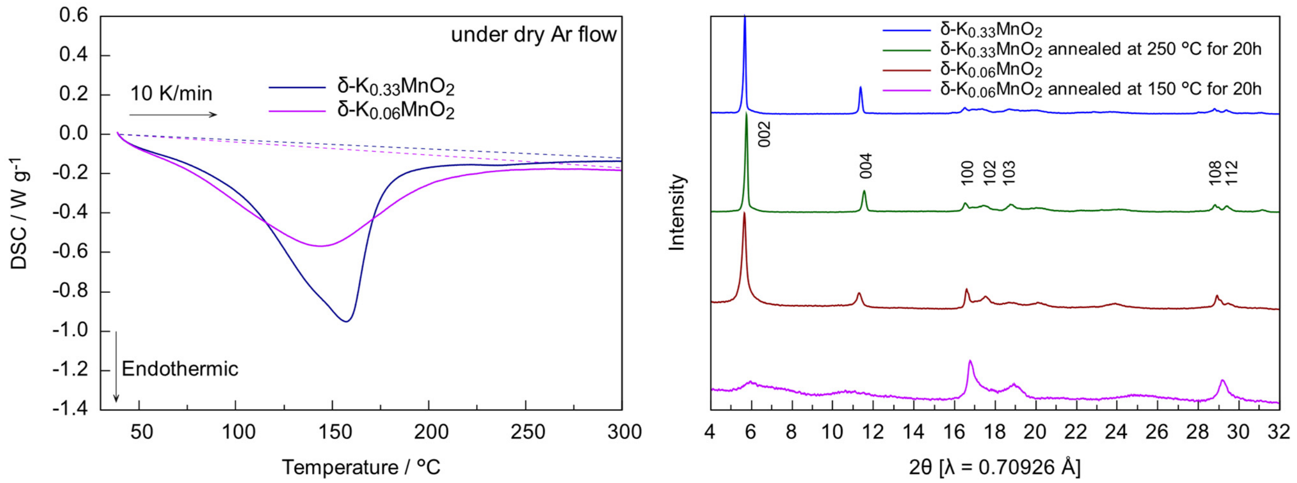

Supplementary Figure 7. Comparative experiments of differential scanning calorimetry (DSC) and ex-situ X-ray diffraction measurements upon heat treatment using powder samples of $K_{0.33}MnO_2$ and $K_{0.06}MnO_2$. In the DSC measurements, the endothermic heat is evaluated to be about 274 kJ per kg-pristine, which is considerably smaller than that (373 kJ per kg-pristine) for $K_{0.33}MnO_2$.

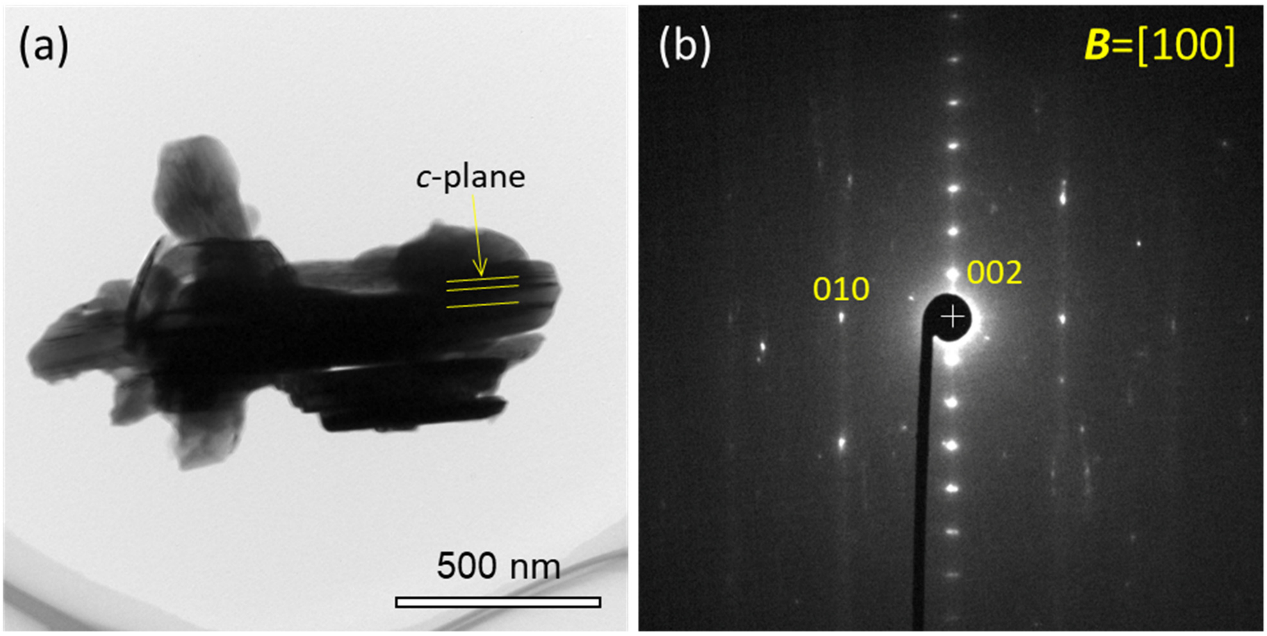

$$\text{The axial ratio: } \frac{c}{a} = \frac{g_{002}^{-1}\times 2}{g_{010}^{-1}\times 2/\sqrt{3}} = \frac{12.63}{2.82} = 4.48$$

Supplementary Figure 8. TEM observation for as-prepared sample. (a) Bright field image and (b) electron diffraction pattern. The TEM sample shows a *c*/*a* value (4.48) close to that for the dehydrated phase (4.57) rather than that of hydrated phase (4.99), which were determined by the in-situ XRD analysis (Fig. 3). Thus, even though we prepared the hydrated sample initially, the sample changed to a fully dehydrated state when we observed this in a vacuum condition of TEM.

Supplementary References

1. Chen, D. *et al.* Probing the Charge Storage Mechanism of a Pseudocapacitive MnO_2 Electrode Using in Operando Raman Spectroscopy. *Chem. Mater.* **27**, 6608-6619 (2015).
2. Morgan Chan, Z. *et al.* Electrochemical trapping of metastable Mn^{3+} ions for activation of MnO_2 oxygen evolution catalysts. *Proc. Natl. Acad. Sci. U.S.A.* **115**, E5261-E5268 (2018).
3. Julien, C. Raman spectra of birnessite manganese dioxides. *Solid State Ion.* **159**, 345-356 (2003).
4. Julien, C. & Massot, M. Spectroscopic studies of the local structure in positive electrodes for lithium batteries. *Phys. Chem. Chem. Phys.* **4**, 4226-4235 (2002).
5. Julien, C. M., Massot, M. & Poinsignon, C. Lattice vibrations of manganese oxides. *Spectrochim. Acta A Mol. Biomol. Spectrosc.* **60**, 689-700 (2004).
